# Validation and Interpretation of the Persian Version of the Swallowing Disturbance Questionnaire in Patients with Multiple Sclerosis

**DOI:** 10.3390/neurosci6040111

**Published:** 2025-11-03

**Authors:** Omid Mirmosayyeb, Mohammad Mohammadi, Saeed Vaheb, Aysa Shaygannejad, Aynaz Mohammadi, Vahid Shaygannejad

**Affiliations:** 1 Isfahan Neurosciences Research Center, Isfahan University of Medical Sciences, Isfahan 8174673461, Iran; omid.mirmosayyeb@uvmhealth.org (O.M.); saeedvaheb@res.mui.ac.ir (S.V.); a.shaygannejad@res.mui.ac.ir (A.S.); 2 Department of Neurology, School of Medicine, Isfahan University of Medical Sciences, Isfahan 8174673461, Iran; 3 School of Medicine, Iran University of Medical Sciences, Tehran 1449614535, Iran; mohammadi.moham@iums.ac.ir (M.M.); mohammadi.a@iums.ac.ir (A.M.)

**Keywords:** multiple sclerosis, dysphagia, swallowing disturbance questionnaire, swallowing

## Abstract

Background: Patients with multiple sclerosis (PwMS) frequently experience dysphagia, which affects their quality of life. The swallowing disturbance questionnaire (SDQ) has demonstrated potential in screening dysphagia in different disorders. The objective of this study was to evaluate the validity and reliability of the Persian version of SDQ in PwMS. Methods: In this cross-sectional study, 198 PwMS were enrolled. The translation of SDQ into Persian was performed using the forward–backward method. Participants completed both the SDQ and the Dysphagia in Multiple Sclerosis (DYMUS) questionnaires. Convergent validity was assessed using the Spearman correlation, construct validity was evaluated by principal component analysis (PCA), and reliability was assessed by Cronbach’s alpha. Screening ability was evaluated with receiver operating characteristic (ROC) curve analysis, using DYMUS as the reference measure. Results: The Persian SDQ showed high internal consistency (Cronbach’s alpha = 0.913) after removing one item. PCA revealed a single dominant factor accounting for 49.4% of the variance. The 14-item SDQ correlated strongly with both DYMUS (Spearman’s rho = 0.62, *p* < 0.001) and Expanded Disability Status Scale (EDSS) (Spearman’s rho = 0.388, *p* < 0.001). The area under the curve of 0.957 revealed high screening power with a sensitivity of 91.7% and a specificity of 88.9%. Conclusions: The Persian SDQ is a valid and reliable tool for early detection and quick monitoring of dysphagia in PwMS.

## 1. Introduction

Multiple sclerosis (MS) is a chronic neurological disorder characterized by the loss of myelin in the central nervous system, which often leads to physical disabilities. In addition to physical symptoms, many patients experience other problems that are not routinely assessed in clinical practice. These invisible symptoms may include cognitive impairment, persistent fatigue, mood changes, pain (both physical and emotional), and issues with bladder, bowel, or sexual function [1]. Such issues can significantly interfere with daily activities and overall quality of life (QoL), sometimes even more than the degree of physical disability measured by the Expanded Disability Status Scale (EDSS) [1]. One of the overlooked symptoms is dysphagia, which can affect more than one-third of patients with MS (PwMS) [2,3]. Poorjavad et al. (2010) reported that problems in the pharyngeal phase of swallowing are more frequent than those in the oral phase among PwMS (28.7% vs. 5%) [4].

Dysphagia presents with significant physical and psychological health challenges in PwMS [5]. Complications may include aspiration pneumonia, choking, malnutrition, and dehydration, as well as reduced QoL caused by social withdrawal, depression, and diminished self-confidence [5,6]. To minimize these risks, it is essential to identify swallowing difficulties in PwMS at an early stage. Although videofluoroscopic swallowing studies (VFSS), fiberoptic endoscopic evaluation of swallowing (FEES), and diagnostic ultrasonography are considered the gold standard for diagnosing dysphagia [7,8,9], their use is frequently limited by radiation exposure, the need for technicians, and their high cost. Standardized screening instruments, such as the Dysphagia in Multiple Sclerosis (DYMUS) questionnaire [10] is a validated and cheap alternative, which includes ten self-administered items (three questions about liquids and seven questions about solids) [11]. Each Yes/No question scores 0 or 1, giving a total score ranging from 0 to 10. Clinically significant dysphagia is indicated by a score of three or higher. With a high internal consistency (Cronbach’s alpha = 0.88), the DYMUS questionnaire is considered a reliable, validated screening tool applicable in clinical and research settings [11].

The DYMUS questionnaire has several limitations that need to be acknowledged. It ignores salivary-related swallowing difficulties and ignores issues with different food textures, like those in pureed foods. Furthermore, the frequency and severity of symptoms cannot be adequately assessed by its Yes/No response format. Some of these limitations have been addressed by the swallowing disturbance questionnaire (SDQ), a thorough 15-item test that investigates both oral and pharyngeal-phase swallowing difficulties. In contrast to DYMUS, the SDQ rates the frequency of symptoms on a 4-point scale: 0 denotes “never,” 1 “rarely” (at least once a month), 2 “often” (one to seven times a week), and 3 “very often” (more than seven times a week) [12]. The revised 14-item Italian SDQ version has proven to be internally consistent, reliable, and clinically relevant in PwMS, offering a more thorough screening tool for swallowing disorders in this population [12]. However, it is essential to acknowledge that higher SDQ scores indicate a higher risk of oropharyngeal dysphagia, but do not necessarily reflect its severity, as this tool is primarily designed for dysphagia screening.

The aim of this study was to evaluate the validity and reliability of the Persian version of SDQ for the assessment of swallowing disturbances in PwMS.

## 2. Materials and Methods

### 2.1. Study Population

In this cross-sectional study, PwMS from the outpatient MS clinic of Kashani Hospital, Isfahan, were enrolled between April 2024 and October 2024. The study’s inclusion criteria were: (1) a confirmed MS diagnosis based on the revised McDonald criteria 2017 [13]; (2) age > 18 years; and (3) voluntary participation; patients with other neurological diseases and conditions causing swallowing problems were excluded. Also, individuals who had previously received therapy for dysphagia were excluded.

### 2.2. Translation of SDQ

To develop the Persian version of the SDQ, a forward–backward translation was done to establish cross-cultural adaptation. The translation process consisted of the following steps:

#### 2.2.1. Forward Translation

Two translators with expertise in medicine independently translated the original English version of the SDQ [12] into the Persian language. The translators were fluent in both Persian and English.

#### 2.2.2. Backward Translation

The Persian translations were then independently back-translated into English by two other translators who had not seen the original SDQ before. These translators were fluent in English and Persian and had experience translating medical textbooks.

#### 2.2.3. Comparison and Reconciliation

The original and back-translated versions of the SDQ were evaluated by a group of neurologists and neurology researchers who gave the translators feedback on their conceptual, semantic, and linguistic equivalency.

#### 2.2.4. Expert Panel Review

The Persian version of the SDQ was assessed by a group of specialists that included linguists and clinicians. The SDQ’s Persian translation was evaluated to make sure it remained conceptually consistent with the original version of the SDQ, linguistically accurate and fluent, and culturally appropriate. Through discussions, the discrepancies were resolved so that the Persian version of the SDQ accurately represented its original contents.

### 2.3. Data Collection and Tests

Demographic and clinical characteristics, such as age, sex, employment status, years of education, marital status, age at MS onset, MS type, disease-modifying therapies (DMT) used by the participants, EDSS [14], and disease duration were collected from all participants’ medical records and confirmed with them during their interview. Additionally, each participant completed the following neuropsychological assessments:

#### 2.3.1. Swallowing Disturbance Questionnaire (SDQ)

The SDQ was originally established to evaluate swallowing difficulties in individuals with Parkinson’s disease (PD). It has an 81.3% specificity and an 80.5% sensitivity [12], and consists of 14 items scored on a 4-point categorical scale: Zero means “never,” one means “rarely” (≥once a month), two means “often” (1–4 times a week), and three means “very often” (>7 times a week). Another question has a Yes or No response, with Yes receiving a score of 2.5 and “No” receiving a score of 0.5. Higher scores indicate higher levels of dysphagia; the total score ranges from 0 to 44.5. With an area under the curve (AUC) of 81.1%, SDQ was recently validated in Italian PwMS and demonstrated as a good screening tool for dysphagia in PwMS [15].

#### 2.3.2. Dysphagia in Multiple Sclerosis (DYMUS)

The 10-item self-administered DYMUS questionnaire was created basically to assess dysphagia in PwMS. It has three items about liquids and seven items about solid foods swallowing problems (Bergamaschi et al., 2008) [11]. Answers are marked as Yes or No, and the sum of the scores ranges from 0 to 10. A score of ≥3 is considered the threshold for dysphagia. In our study, the reference screening tool for dysphagia in PwMS was the Persian version of the DYMUS [16].

#### 2.3.3. Multiple Sclerosis Impact Scale (MSIS-29)

The MSIS-29 is a self-report questionnaire with 29 items that assesses how MS has impacted a person’s daily life in both physical and psychological ways over the past two weeks. A 5-point Likert scale is used to score each item. The MSIS-29 is divided into two subscales: a psychological scale with nine items and a physical scale with twenty items. The Persian version of MSIS-29 [17] was used in this study.

### 2.4. Data Analysis

Categorical variables were presented as counts and percentages (%), and continuous variables were summarized using the median and interquartile range (IQR).

To assess the reliability of the Persian SDQ, internal consistency was measured through Cronbach’s alpha. A Cronbach’s alpha above 0.70 and an item-total correlation greater than 0.30 were considered acceptable for each item [18,19]. Items that did not meet either of these requirements were excluded from the questionnaire.

Principal component analysis (PCA) was used to assess the construct validity of the SDQ. The data’s suitability for factor analysis was confirmed by Bartlett’s test of sphericity and the Kaiser-Meyer-Olkin (KMO) measure of sampling adequacy. To perform PCA, Bartlett’s test had to be significant, and the KMO value had to be greater than 0.6 [20]. An initial PCA with oblimin rotation was then conducted to explore the factor structure of the SDQ. To determine the number of factors, two criteria were used: the eigenvalue rule (retaining factors with eigenvalues greater than 1) and parallel analysis. Parallel analysis involved comparing the unrotated eigenvalues derived from a randomly generated dataset with the same number of cases and variables as the actual data. Factors were retained as significant if their eigenvalues from the PCA surpassed those obtained from the parallel analysis. Questionnaire items with factor loadings higher than 0.3 were considered acceptable and retained [21,22,23].

To assess the convergent validity of the SDQ, Spearman correlation analysis was performed between the final version of the SDQ and DYMUS. Additionally, correlations were assessed between the SDQ and MSIS-29, as well as clinical and demographic variables. To evaluate the screening ability of the SDQ, a receiver operating characteristic (ROC) analysis was performed. The DYMUS test, with a cutoff score of ≥3 for dysphagia, served as the reference screening tool [24]. All statistical analyses were conducted via IBM SPSS version 26.0 (IBM Corp., Armonk, NY, USA).

## 3. Results

### 3.1. Study Population Characteristics

A total of 198 PwMS were included in this validation study. The median age of participants was 36 years (IQR: 30–40), the majority of whom (91.4%) had relapsing-remitting MS (RRMS). 86.4% of participants were female, and 54.5% of them were not employed. The median disease duration among participants was 7 years (IQR: 4–10), and the median EDSS was 1 (IQR: 1–2.5). Notably, dysphagia was diagnosed in 18.2% of the patients based on the DYMUS questionnaire. Detailed demographic and clinical characteristics of the study population are presented in Table 1.

### 3.2. Reliability

Table 2 presents the SDQ internal consistency analysis using Cronbach’s alpha. For the 15-item SDQ, the overall Cronbach’s alpha was 0.892, showing good internal consistency. However, Question 15 demonstrated a notably low corrected item-total correlation (0.124) and a significant increase in Cronbach’s alpha (to 0.913) when the item was removed. This finding supports the exclusion of Question 15, resulting in the revised 14-item SDQ.

The updated 14-item SDQ showed better internal consistency, with an overall Cronbach’s alpha of 0.913. The 14-item SDQ’s corrected item-total correlations ranged from 0.338 to 0.763, indicating acceptable to strong relationships between individual items and the overall score.

### 3.3. Construct Validity

Bartlett’s test of sphericity was used to determine whether the data were suitable for factor analysis, and the results were significant (χ^2^ = 1510.791, df = 91, *p* < 0.001). This indicates that variances differed across components and that correlations between items were sufficiently strong to proceed with PCA. Additionally, the sample size was confirmed to be adequate for factor analysis with a KMO value of sampling adequacy of 0.903.

**Figure 1:**
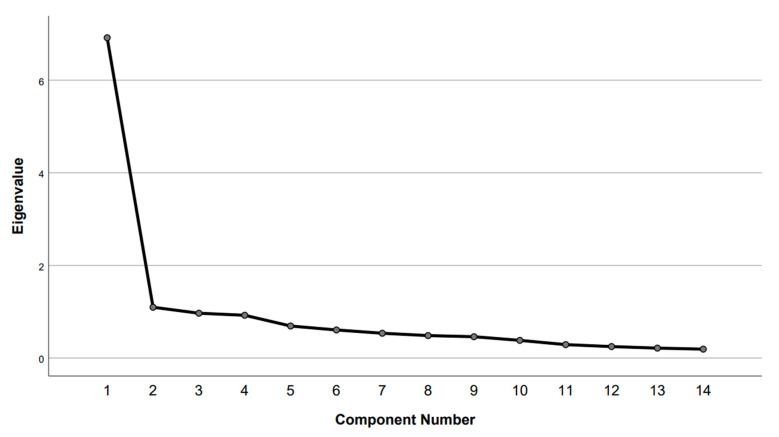
Scree plot.

Initial PCA identified two factors for the 14-item SDQ, with eigenvalues of 6.916 for the first factor and 1.096 for the second factor. The scree plot (Figure 1) visually supports these findings. The first factor accounted for 49.4% of the total variance, while the second factor accounted for 7.8%. Parallel analysis revealed that the eigenvalue of the first factor was significantly greater than what would be expected by chance. In contrast, the eigenvalue for the second factor was lower than that of a random dataset. Consequently, the second factor was excluded.

All 14 items demonstrated factor loadings greater than 0.3 for the first factor, confirming their relevance and retention in the questionnaire. The distribution of responses from PwMS to the SDQ questions, along with the factor loadings from PCA, is summarized in Table 3.

### 3.4. Convergent Validity and Other Correlations

The results showed a strong positive correlation between the 14-item SDQ and DYMUS scores (Spearman’s ρ = 0.62, *p* < 0.001), indicating strong convergent validity. Additionally, the 14-item SDQ was moderately correlated with EDSS scores (Spearman’s ρ = 0.388, *p* < 0.001), further supporting its clinical relevance.

Among demographic variables, a weak but significant negative correlation was found between the 14-item SDQ and education level (Spearman’s ρ = −0.166, *p* = 0.019), while the correlation with age was not statistically significant (Spearman’s ρ = 0.136, *p* = 0.057). Other clinical and demographic variables, including age at onset and disease duration, did not show significant correlations with the 14-item SDQ.

In terms of QoL measures, the 14-item SDQ had a weak but significant negative correlation with the physical scale of the MSIS-29 (Spearman’s ρ = −0.170, *p* = 0.017). However, no significant correlation was found between the 14-item SDQ and the psychological scale of the MSIS-29 (Spearman’s ρ = −0.014, *p* = 0.847). The detailed results of these correlations are presented in Table 4.

### 3.5. Interpretability

**Figure 2:**
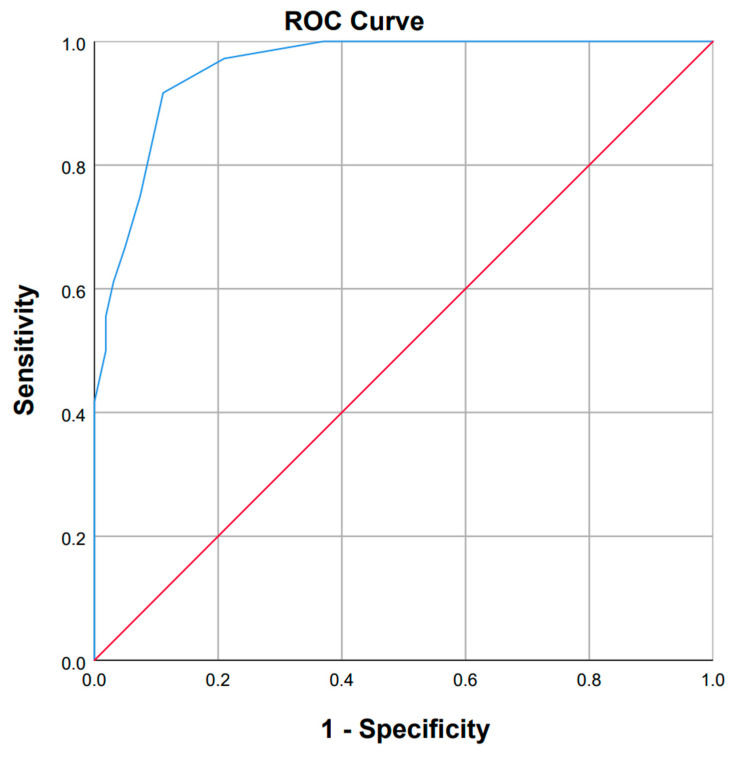
ROC curve for the 14-item Persian SDQ against the DYMUS questionnaire as the reference tool. The blue line represents the ROC curve of the Persian SDQ. The red line indicates the line of no discrimination (AUC = 0.5).

ROC curve analysis was used to evaluate the 14-item SDQ’s interpretability (Figure 2), using the DYMUS questionnaire as the reference screening instrument. With an AUC of 0.957, a sensitivity of 91.7%, and a specificity of 88.9% for SDQ, the analysis showed outstanding agreement with the DYMUS. These findings demonstrate how well the 14-item SDQ can screen dysphagia in PwMS.

## 4. Discussion

According to this study, the Persian 14-item SDQ is a reliable and valid tool for assessing dysphagia in PwMS. The findings showed strong construct validity and excellent internal consistency (Cronbach’s alpha = 0.913). PCA showed that 49.4% of the variance was explained by a single dominant factor. Significant correlations were also found with the DYMUS questionnaire and the EDSS, supporting convergent validity. With an AUC of 0.957, a sensitivity of 91.7%, and a specificity of 88.9%, the SDQ revealed strong screening performance. These findings underline the effectiveness of the Persian 14-item SDQ for clinical and research settings in PwMS.

The SDQ is an established tool that is known for its adaptability and reliability in screening dysphagia in a variety of medical conditions. Studies have demonstrated that the SDQ is useful in identifying swallowing problems in PD patients, with 80.5% sensitivity and 81.3% specificity, when compared to objective tests like the FEES, despite the fact that it was first developed and validated for this population [12]. Studies further support its broader clinical relevance, showing that the SDQ can be used to evaluate dysphagia not only in PD but also in stroke and other neurological disorders [25,26,27].

Sparaco et al. (2024) validated the Italian version of the 14-item SDQ to assess dysphagia in PwMS [15]. This study, which showed an AUC of 81.1%, is consistent with our results [15]. Language and cultural differences, dietary habits, or healthcare access between the Italian and Persian MS populations and variations in the study populations may have contributed to the marginally better screening ability seen in our study when compared to the Italian version. For example, certain food textures common in the Persian diet, such as rice-based dishes or thicker stews, might elicit more noticeable swallowing difficulties than foods typically consumed in Italy, potentially influencing swallowing assessments. These differences should be explored in future cross-cultural validation studies. In addition, our results support the findings of Sparaco et al. by confirming a significant positive correlation between the EDSS and 14-item SDQ scores [15]. The correlation between EDSS and DYMUS scores, a particular MS questionnaire, was also reported by Bergamaschi et al. (r = 0.22, *p* = 0.0007) [15]. This strengthens the possible connection between dysphagia and disability in MS. However, no significant correlation was found between 14-item SDQ scores and either disease duration or age [15].

To our knowledge, no previous study has examined the relationship between the 14-item SDQ and the MSIS-29. Our findings indicate a weak but statistically significant negative correlation between the 14-item SDQ and the physical component of the MSIS-29 (Spearman’s ρ = −0.170, *p* = 0.017), suggesting that although a higher perceived physical burden of MS is linked to greater swallowing difficulties, the relatively mild level of disability in our MS group (median EDSS = 1) may explain the weak correlation between SDQ scores and the physical component of MSIS-29, as swallowing difficulties may have a less pronounced impact on QoL in less disabled individuals. However, no significant correlation was found between the 14-item SDQ and the psychological component (Spearman’s ρ = −0.014, *p* = 0.847). These results may emphasize the specific impact of physical QoL on swallowing disturbances in PwMS rather than having a psychological reason, highlighting the need for targeted interventions addressing physical disability.

Our findings highlight the importance of early dysphagia screening in PwMS, particularly those with higher disability. As there is a notable link between physical impairment and swallowing problems, regular dysphagia assessment may lead to timely interventions and improved QoL in patients. According to a study by Tahir et al., dysphagia in PwMS may lower their QoL [15]. Furthermore, dysphagia risk assessment can be enhanced by evaluating the presence of voice changes following swallowing, which is assessed in the 14-item SDQ but not in DYMUS. According to a systematic review by Dos Santos et al. (2022), voice changes that occur after swallowing could be a sign of dysphagia risk [28]. Given its strong screening ability, the Persian 14-item SDQ could be incorporated into standard MS assessments as an initial screening tool, particularly for patients with moderate to severe disability. However, when there is significant swallowing dysfunction or diagnostic uncertainty, objective tests such as the VFSS or FEES should still be considered. Additionally, assessing patients’ stated difficulties with solids, liquids, and pureed foods can also help PwMS with early intervention and dietary management.

Although Item 15, which assesses dysphagia complications such as pneumonia, is clinically relevant, it was omitted from the Persian version due to its weak statistical performance. This approach improved the internal consistency of the questionnaire. We suggest that future studies with larger sample sizes and in different cultural contexts further investigate this item.

When evaluating the findings, it is critical to consider the limitations of the current study. First, a significant limitation of this study is its reliance on patient-reported outcomes rather than instrumental or clinician-rated dysphagia assessments (e.g., VFSS, FEES). As a result, the reported screening performance of the Persian SDQ reflects agreement with another validated questionnaire rather than accurate diagnostic accuracy for clinically verified dysphagia or aspiration risk. Future studies should validate the SDQ against objective dysphagia measures to confirm its screening ability. When compared to clinical evaluations, prior research has shown that the SDQ has high sensitivity and specificity. For instance, compared with FEES, the SDQ demonstrated 87.6% specificity and 85.71% sensitivity [25]. When compared to VFSS in PD patients, it showed 77.8% sensitivity and 84.6% specificity in a study in Japan [29]. Comparing the Persian version of the SDQ to the VFSS in PD patients, it was found to have 96.7% sensitivity and 91.2% specificity [30]. But compared to VFSS, the SDQ’s sensitivity (28.57%) and specificity (68.18%) in identifying penetration/aspiration and dysphagia in early and middle-stage PD patients were inadequate, according to a recent study by Ponsoni et al. [31]. These results emphasize the importance of using objective clinical tools alongside the SDQ in future studies with more cases to clarify its accuracy compared to objective tools. Second, due to the relatively low disability levels in our cohort, the utility and psychometric properties of the Persian SDQ should be further confirmed in populations with higher disability, where dysphagia screening is especially critical. At the same time, demonstrating good screening accuracy in patients with lower EDSS is a strength, as it suggests the SDQ can help identify swallowing difficulties earlier in the disease course. Another limitation is that most participants in our cohort had few swallowing problems, which may limit the generalizability of the findings to PwMS with severe dysphagia. Third, due to the limited availability of sources, our study did not conduct a test–retest analysis. Finally, because over 86% of participants were female, the results might not be entirely applicable to the male MS population, which would restrict their broader applicability.

## 5. Conclusions

In summary, the Persian version of the 14-item SDQ proved to be a reliable, valid, and interpretable tool for dysphagia screening in MS. In turn, earlier detection of swallowing issues could prompt interventions (diet modifications, swallowing therapy) that prevent serious complications like aspiration pneumonia. To assess the 14-item SDQ’s suitability for various MS subgroups, studies should examine its validity across a range of populations, taking into account factors such as MS subtype, disease severity, and sex. Furthermore, longitudinal studies are recommended to evaluate the condition’s long-term implications on clinical outcomes and QoL in patients with different MS subtypes. This will help us better understand the progressive effects of dysphagia.

## Figures and Tables

**Table 1:** Demographic and clinical characteristics of participants.

Variables		PwMS (n = 198)
Age, median (IQR)		36 (30–40)
Gender, n (%)	Female	171 (86.4)
	Male	27 (13.6)
Marital status, n (%)	Single	51 (25.8)
	Married	147 (74.2)
Education years, median (IQR)		16 (12–16)
Job, n (%)	Not occupied	108 (54.5)
	Occupied	90 (45.5)
MS type, n (%)	RRMS	181 (91.4)
	SPMS	14 (7.1)
	PPMS	3 (1.5)
Age at onset, median (IQR)		27 (22.9–32)
Disease duration, median (IQR)		7 (4–10)
EDSS, median (IQR)		1 (1–2.5)
DMT, n (%)	RTX	68 (34.3)
	DMF	47 (23.7)
	IFN	28 (14.1)
	TFN	28 (14.1)
	OCR	22 (11.1)
	Fingolimod	3 (1.5)
	GA	2 (1)
DYMUS, median (IQR)		1 (0–2)
	No dysphagia, n (%)	162 (81.8)
	Dysphagia, n (%)	36 (18.2)
SDQ-15 item, median (IQR)		1.5 (0.5–3.5)
SDQ-14 item, median (IQR)		0 (0–3)
MSIS-29 (psychological scale), median (IQR)		58.3 (44.4–66.7)
MSIS-29 (physical scale), median (IQR)		63.8 (56.3–71.3)

PwMS: patients with multiple sclerosis; n: number of participants; SD: standard deviation; IQR: interquartile range; MS: multiple sclerosis; RRMS: relapsing-remitting MS; PPMS: primary progressive MS; SPMS: secondary progressive MS; EDSS: expanded disability status scale; DMT: disease modifying therapy; RTX: Rituximab; DMF: Dimethyl Fumarate; IFN: Interferon; TFN: Teriflunomide; OCR: Ocrelizumab; GA: Glatiramer acetate; DYMUS: Dysphagia in Multiple Sclerosis; SDQ: swallowing disturbance questionnaire; MSIS-29: Multiple Sclerosis Impact Scale.

**Table 2:** Internal consistency using standard Cronbach’s alpha analysis.

SDQ Questions	15-Item SDQ		14-Item SDQ	
	Corrected Item-Total Correlation	Cronbach’s Alpha if Item Deleted	Corrected Item-Total Correlation	Cronbach’s Alpha if Item Deleted
Q1	0.75	0.877	0.763	0.901
Q2	0.624	0.883	0.637	0.906
Q3	0.345	0.893	0.338	0.915
Q4	0.531	0.887	0.529	0.91
Q5	0.566	0.887	0.579	0.912
Q6	0.66	0.882	0.667	0.905
Q7	0.733	0.879	0.752	0.902
Q8	0.6	0.887	0.61	0.909
Q9	0.683	0.881	0.702	0.904
Q10	0.647	0.882	0.648	0.906
Q11	0.721	0.88	0.725	0.903
Q12	0.708	0.881	0.688	0.905
Q13	0.539	0.887	0.549	0.911
Q14	0.709	0.881	0.725	0.904
Q15	0.124	0.913	-	-
Cronbach alpha (overall)	0.892		0.913	

SDQ: swallowing disturbance questionnaire.

**Table 3:** SDQ: number and percentages of total answers scoring between 0 and 3 for each question, along with factor loadings from PCA.

Item Number	Item Content	n (%) of Answers for Each Score				Factor Loadings
		0	1	2	3	
1	Do you experience difficulty chewing solid food like an apple, cookie or a cracker?	163 (82.3)	26 (13.1)	8 (4)	1 (0.5)	0.802
2	Are there any food residues in your mouth, cheeks, under your tongue or stuck to your palate after swallowing	167 (84.3)	24 (12.1)	6 (3)	1 (0.5)	0.798
3	Does food or liquid come out of your nose when you eat or drink?	188 (94.9)	8 (4)	2 (1)	0 (0)	0.789
4	Does chewed up food dribble from your mouth?	177 (89.4)	17 (8.6)	4 (2)	0 (0)	0.783
5	Do you feel you have too much saliva in your mouth; do you drool or have difficulty swallowing your saliva?	161 (81.3)	22 (11.1)	10 (5.1)	5 (2.5)	0.77
6	Do you swallow chewed up food several times before it goes down your throat?	174 (87.9)	17 (8.6)	6 (3)	1 (0.5)	0.747
7	Do you experience difficulty in swallowing solid food (i.e., do apples or crackers get stuck in your throat)?	174 (87.9)	19 (9.6)	4 (2)	1 (0.5)	0.724
8	Do you experience difficulty in swallowing pureed food?	188 (94.9)	8 (4)	2 (1)	0 (0)	0.704
9	While eating, do you feel as if a lump of food is stuck in your throat?	173 (87.4)	19 (9.6)	5 (2.5)	1 (0.5)	0.701
10	Do you cough while swallowing liquids?	155 (78.3)	38 (19.2)	4 (2)	1 (0.5)	0.684
11	Do you cough while swallowing solid foods?	175 (88.4)	19 (9.6)	3 (1.5)	1 (0.5)	0.634
12	Immediately after eating or drinking, do you experience a change in your voice, such as hoarseness or reduced?	179 (90.4)	15 (7.6)	3 (1.5)	1 (0.5)	0.609
13	Other than during meals, do you experience coughing or difficulty breathing as a result of saliva entering your windpipe?	153 (77.3)	34 (17.2)	9 (4.5)	2 (1)	0.593
14	Do you experience difficulty in breathing during meals?	181 (91.4)	13 (6.6)	3 (1.5)	1 (0.5)	0.382
15	Have you suffered from a respiratory infection (pneumonia, bronchitis) during the past year?	No: 168 (84.8)		30 (15.2)		-

PCA: principal component analysis; SDQ: swallowing disturbance questionnaire; n: number of participants.

**Table 4:** Correlation between SDQ and DYMUS, MSIS-29, clinical, and demographic variables.

		SDQ
Age	Spearman’s rho	0.136
	*p*-value	0.057
Education	Spearman’s rho	−0.166
	*p*-value	0.019
Age at onset	Spearman’s rho	0.071
	*p*-value	0.323
Disease duration	Spearman’s rho	0.055
	*p*-value	0.441
EDSS	Spearman’s rho	0.388
	*p*-value	<0.001
DYMUS	Spearman’s rho	0.62
	*p*-value	<0.001
MSIS-29 (psychological scale)	Spearman’s rho	−0.014
	*p*-value	0.847
MSIS-29 (physical scale)	Spearman’s rho	−0.170
	*p*-value	0.017

DYMUS: Dysphagia in Multiple Sclerosis; SDQ: swallowing disturbance questionnaire; MSIS-29: Multiple Sclerosis Impact Scale; EDSS: Expanded Disability Status Scale.

## Data Availability

The data presented in this study are available on request from the corresponding author due to ethical reasons.
